# Analyzing Cold Tolerance Mechanism in Transgenic Zebrafish (*Danio rerio*)

**DOI:** 10.1371/journal.pone.0102492

**Published:** 2014-07-24

**Authors:** Qian Wang, Xungang Tan, Shuang Jiao, Feng You, Pei-Jun Zhang

**Affiliations:** 1 Key Laboratory of Experimental Marine Biology, Institute of Oceanology, Chinese Academy of Sciences, Qingdao, Shandong, China; 2 University of Chinese Academy of Sciences, Beijing, China; Karlsruhe Institute of Technology, Germany

## Abstract

Low temperatures may cause severe growth inhibition and mortality in fish. In order to understand the mechanism of cold tolerance, a transgenic zebrafish *Tg* (*smyd1:m3ck*) model was established to study the effect of energy homeostasis during cold stress. The muscle-specific promoter *Smyd1* was used to express the carp muscle form III of creatine kinase (*M3*-*CK*), which maintained enzymatic activity at a relatively low temperature, in zebrafish skeletal muscle. *In situ* hybridization showed that *M3*-*CK* was expressed strongly in the skeletal muscle. When exposed to 13°C, *Tg* (*smyd1:m3ck*) fish maintained their swimming behavior, while the wild-type could not. Energy measurements showed that the concentration of ATP increased in *Tg* (*smyd1:m3ck*) versus wild-type fish at 28°C. After 2 h at 13°C, ATP concentrations were 2.16-fold higher in *Tg* (*smyd1:m3ck*) than in wild-type (*P*<0.05). At 13°C, the ATP concentration in *Tg* (*smyd1:m3ck*) fish and wild-type fish was 63.3% and 20.0%, respectively, of that in wild-type fish at 28°C. Microarray analysis revealed differential expression of 1249 transcripts in *Tg* (*smyd1:m3ck*) versus wild-type fish under cold stress. Biological processes that were significantly overrepresented in this group included circadian rhythm, energy metabolism, lipid transport, and metabolism. These results are clues to understanding the mechanisms underlying temperature acclimation in fish.

## Introduction

Water temperature is considered a major environmental factor influencing the development, growth, reproduction, and behavior of aquatic ectotherms [1], [2]. Fish, one kind of aquatic ectotherm, may face a large range of daily or seasonal temperature fluctuations [3]. One way to cope with these challenges is to migrate to areas with more suitable temperatures [4]. Another way is to evolve the ability to acclimate to environmental temperature fluctuations. Many species have evolved a variety of mechanisms to alter cellular biochemistry, such as by producing temperature-specific isoenzymes [5], changing the membrane lipid content and the degree of fatty acid unsaturation [6], [7], recruiting different muscle fiber types [8], inducing molecular chaperones [9], and generating antifreeze proteins during long-term evolutionary adaptation to a freezing environment [10]. These provide clues to the mechanism by which fish cope with temperature changes and enhance their cold tolerance.

These “thermal compensations” may rely on energy metabolism to carry out every kind of physical activity. Muscle occupies more than 75% of the whole body. Muscle contraction is the main reaction by which chemical energy is converted to mechanical work and heat, the latter being essential for maintaining body temperature [11], [12]. Muscle contraction requires large amounts of ATP, most of which is derived from mitochondrial electron transport and oxidative phosphorylation of substrates from fatty acid or glucose metabolism. When high-energy supplementation is needed, ATP can be complemented by phosphocreatine generated by creatine kinase. Creatine kinase (CK, EC 2.7.3.2) belongs to the phosphagen kinase family and functions in chemical energy storage and quick release; it is thus important for energy metabolism in muscle. CK removes a phosphate from ATP to produce ADP and phosphocreatine; in the reverse reaction, it adds a phosphate to ADP to produce creatine and ATP [13], [14]. In common carp (*Cyprinus carpio*), three sub-isoforms of muscle creatine kinase have been designated M1-CK, M2-CK, and M3-CK [15]. The formation of distinct homo- or heterodimers of M-CKs was altered in response to temperature acclimatization. At high temperature (30°C), M1M1-CK and M2M2-CK homodimers and M1M3-CK heterodimers predominate, whereas M3M3-CK homodimerizes at low temperature (10°C). M3-CK remained stable; it maintained its high enzyme activity and it was thought to be the dominant isoform mediating carp acclimation to lower environmental temperature [16].

The zebrafish (*Danio rerio*) has become one of the most important model organisms in many fields of research including genetics, neuroscience, development, physiology, toxicology, and biomedicine [17], [18]. Zebrafish are a tropical teleost species; the extreme temperatures recorded in known zebrafish habitats were 16.5°C and 38.6°C [19]. A number of other attributes have contributed to the scientific importance to this species, such as its small size, rapid development, and well-known genetic resources. Zebrafish were therefore considered suitable for use as a model for understanding the mechanisms of acclimation under low temperatures.

The purpose of this study was to investigate the mechanism of cold tolerance in a transgenic zebrafish model expressing carp M3-CK driven by the zebrafish muscle-specific *smyd1* promoter. Cold tolerance ability in the transgenic zebrafish *Tg* (*smyd1:m3ck*) was evaluated by measuring swimming performance, balance, orientation at low temperatures, and energy production (ATP concentration). Microarray-based expression profiling was used to compare the whole transcriptome of *Tg* (*smyd1:m3ck*) and wild-type fish after acute low-temperature treatment. A group of genes related to circadian rhythm, energy metabolism, lipid transport, and other metabolic pathways were significantly up-regulated under cold-stress in the transgenic fish. These data provided functional genomic and physiological evidence for cold acclimation in fish.

## Materials and Methods

### Ethics statement

Experiments involving zebrafish were conducted according to the regulations of local and central government, and were approved by the Institutional Animal Care and Use Committee of the Institute of Oceanology, Chinese Academy of Sciences.

### Zebrafish maintenance

The adult captive-bred strain of zebrafish (wild-type) was originally obtained from an Aqua Hobby in Nanshan market (Qingdao, China) and acclimated to laboratory conditions. Wild-type and transgenic fish were reared at 28°C±0.5°C and under a photoperiod regime of 14/10 h light/dark. Water quality was maintained by circulation with a filtration system.

### Transgenic plasmid construction

**Figure 1:**
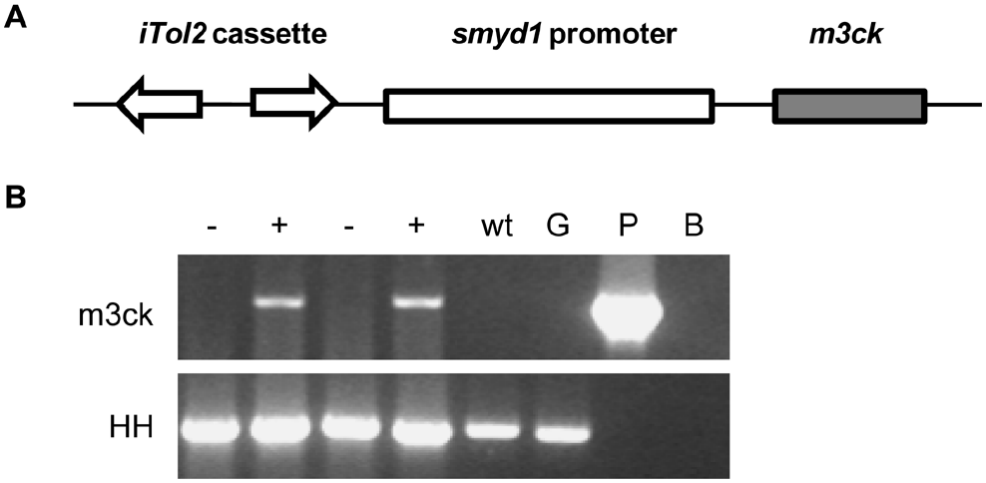
The transgenic cassette and identification of transgenic zebrafish. A. Design of the transgenic cassette. *m3ck* was driven by the skeletal muscle-specific promoter *smyd1* and the *iTol2* cassette was introduced for germline transgenes. B. Identification of transgenic zebrafish by PCR with *m3ck* primers. HH-F/R primers derived from *tiggy winkle hedgehog* exon 3 were used as control primers. −, non-transgenic fish; +, transgenic fish; wt, embryo of wild-type fish; G, genomic DNA of wild-type zebrafish; P, the transgenic plasmid *iTol2*-*smyd1*-*m3ck* as positive control; B, blank control.

The design of the transgenic cassette is illustrated in Figure 1A. The *smyd1*-*gfp* plasmid [20] containing the 5.3-kb *smyd1* promoter fragment was used as the backbone of the transgenic plasmid; the *gfp* cassette was released by *Bam*HI digestion. The 1150-bp carp *m3ck* gene was amplified from the cDNA of common carp by PCR with m3ck-F and m3ck-R primers, according to the archived sequence (GenBank: AF055290.1); the 15-base homologous sequences on each side of the *smyd1*-*gfp Bam*HI site were added to the 5′ end of each primer (Table 1). The *m3ck* cassette was recombined into the *Bam*HI site of *smyd1*-*gfp* (CloneEZ PCR Cloning Kit, GenScript, USA) to obtain the *smyd1*-*m3ck* construct. The *iTol2* cassette [21] was PCR-amplified from *piTol2-Kan* [22] with primers iTol2-F and iTol2-R, in which a *Sal*I site was incorporated at the 5′ end (Table 1). The PCR product was cloned into the *Sal*I site of *smyd1*-*m3ck* to generate the transgenic plasmid *iTol2*-*smyd1*-*m3ck*. PCR cycling conditions for *m3ck* and *iTol2* were as follows: 5 min initial denaturation at 94°C; 30 cycles of denaturation at 94°C for 30 s, annealing at 55°C for 30 s, and extension at 72°C for 1.5 min; and a final 10-min extension at 72°C.

**Table 1:** PCR primers for constructing and identifying transgenic zebrafish.

Primer	Sequence
m3ck-F	5′-**AAGAATTCCTCGACG**CATGACTAAGAACTG-3′
m3ck-R	5′- **GCTCTAGAACTAGTG**GCCATTTTACTTCTG-3′
iTol2-F	5′-GTCGACCCCTGCTCGAGCCGGGCCCAAGTG-3′
iTol2-R	5′-GTCGACATTATGATCCTCTAGAT CAGATCT-3′
HH-F	5′- GGACGGTGACACTTGGTGATG-3
HH-R	5′- CGAGTGGATGGAAAGAGTCTC-3
Tol2L-out	5′-CCCTGCTCGAGCCGGGCCCAAGTG-3′
Tol2R-out	5′-ATTATGATC-CTCTAGATCAGATCT-3′

Homologous sequence was highlighted in bold.

### Generation of transgenic zebrafish

*Tol2* transposase RNA was synthesized *in vitro* using pSP64 poly(A)/*Tol2* transposase plasmid [23]. The plasmid was linearized with *Xba*I, and capped mRNA was synthesized using mMESSAGE mMachine SP6 Kit (Ambion, USA). *iTol2*-*smyd1*-*m3ck* plasmid DNA (50 ng/µL) and Tol2 transposase mRNA (20 ng/µL) were co-injected into one-cell stage zebrafish embryos (wild-type) with PLI-100 Basic Pico-Injector (Harvard, USA). Injected fish were cultured to sexual maturity and outcrossed to identify germline transgenic carriers.

### Identification of transgenic zebrafish

To identify F0 transgenic zebrafish, each putative founder was reared to sexual maturity and outcrossed with wild-type fish to obtain the corresponding F1 progenies. For each founder, 100 F1 embryos were collected at 72 h post-fertilization (hpf) and anesthetized with 100 mg/L MS222 (Tricaine methanesulfonate). The embryos were submerged in 500 µL genomic DNA extraction buffer (10 mM Tris pH 8, 2 mM EDTA, 0.2% Triton X-100, 200 µg/mL Proteinase K) and ground thoroughly. The embryos were incubated at 50°C and vortexed occasionally until completely dissolved. After boiling in a water bath for 10 min, samples were centrifuged at 12,000 rpm for 5 min; the supernatant was stored at −20°C. PCR was performed in a total volume of 25 µL with 1.5 µL supernatant. The m3ck-F/R primers were used to detect transgenic embryos. The HH-F/R primers derived from the *tiggy winkle hedgehog* exon 3 sequence were used for the control [24] (Table 1) with the cycling conditions described above. The transgenic zebrafish were named *Tg* (*smyd1:m3ck*).

The F1 generation was produced by outcrossing F0 with wild-type; the F2 generation was produced by an inter-cross of F1 progeny. To identify transgenic zebrafish of the F1 and F2 generation, fish were cultured to age two months. Half of the tail fin was cut and sampled as above using 40 µL of genomic DNA extraction buffer. PCR was performed as described above.

### Whole-mount *in situ* hybridization

To detect muscle-specific *m3ck* expression, whole-mount *in situ* hybridization was performed on the corresponding F1 transgenic fish. The F0 *Tg* (*smyd1:m3ck*) was crossed with wild-type to produce an F1 generation. The F1 embryos were raised to 72 hpf and anesthetized with 100 mg/L MS222, fixed in 4% paraformaldehyde/PBS overnight at 4°C, and bleached with bleaching buffer [according to the Zebrafish Book, 5th Edition, Westerfield (2007)]. The embryos were washed in PBST, transferred to methanol, and stored at −20°C. Whole-mount *in situ* hybridization with a digoxigenin (DIG)-labeled antisense RNA probe was performed as described [25]. The carp *m3ck* PCR product was cloned into the pBluescript II SK (+) *Sal*I site to generate the *m3ck*-*BSSK* fusion construct. The *m3ck*-*BSSK* plasmid was linearized with *Eco*RI and used to synthesize the riboprobe by using a DIG RNA Labeling Kit (Roche, Germany) by *in vitro* transcription with T3 RNA polymerase. Immunoreactive signal was detected by color microscopy.

### Gene insertion site analysis

Inverse PCR was performed to analyze the insertion site in the zebrafish genome. Genomic DNA from transgenic zebrafish was extracted and 3 µg was digested with *Alu*I or *Hae*III for 5 h. After self-ligation with T4 Ligase (Promega, USA) for 24 h at 15°C, circular genomic DNA was obtained and used as the PCR template in a 50-µL reaction containing 200 ng DNA, 0.4 µM Tol2L-out and Tol2R-out primers [21] (Table 1), and 25 µL of 2× Taq Polymerase Mix (CWBIO, China). Cycling conditions were as follows: 5 min initial denaturation at 94°C; 35 cycles of denaturation at 94°C for 30 s, annealing at 52°C for 30 s and extension at 72°C for 2 min; and a 10 min final extension at 72°C. The PCR product was sequenced and compared to the zebrafish genome database to identify the insertion sites.

### Cold stress

The F2 generation was raised to the age of 3 months (2.17±0.02 cm, 0.42±0.01 g) before use in the cold acclamation experiment. Consistent with previous reports [26], 13°C was selected to represent cold stress. Treatment time was determined according to the (1) experimental feasibility; and (2) time required to discern a differential response between groups. Cold stress was applied for 2 h according to Long et al. [27] and our own preliminary experiments. Before cold treatment, the fish were transferred to tanks with small openings in the bottom and allowed to stand for one day. They were gently moved to the cold bath for the experiment. The groups (n = 30 per group) were designated m3ck-13°C, m3ck-28°C, wt-13°C, and wt-28°C. After temperature stress for 2 h, the fish were anesthetized with 100 mg/L MS222. To determine the ATP concentrations, fish were dissected on ice and the muscle was sampled. For microarray or real-time quantitative PCR analysis, the whole fish was transferred immediately into liquid nitrogen.

### Determination of ATP concentration

After cold stress, five fish from each group were sampled. Intracellular ATP levels were determined using a bioluminescence ATP assay kit (Beyotime, China). Approximately 30–40 mg muscle samples were removed from each fish, homogenized in lysis buffer at a final concentration of 10 mg/100 µL, and centrifuged at 12,000×g to collect the supernatant. ATP detection working solution (100 µL) was added to each well of a 96-well white culture plate and incubated for 3–5 min at room temperature. Then 80 µL of lysis muscle solution and 100 µL of luciferin–luciferase reagent were mixed for 3 s before luminescence was measured over 10 s on an Infinite F200 PRO multimode reader (TECAN, USA). Each lysate was examined in three parallel wells. The 96-well plates also contained wells with serial dilutions of an ATP standard to generate a standard curve. Results were expressed as relative ATP levels.

### Microarray analysis

Gene expression in triplicate samples of m3ck-13°C, m3ck-28°C, wt-13°C, and wt-28°C was assessed by Agilent Zebrafish Oligo Microarray 4×44 K (V3) (design ID: 026437) at ShanghaiBio Corporation (SBC). Twelve microarray experiments were performed, each with three fish. When encountering cold stress, fish perform as an integrated system and cold tolerance is judged from the whole-body response. So, RNA samples were extracted from whole zebrafish with Trizol Reagent (Invitrogen, CA, USA). RNA quality as expressed by the RNA integrity number (RIN) was determined on an Agilent Bioanalyzer 2100 (Agilent Technologies, Santa Clara, CA, USA) and then purified with an RNeasy mini kit (QIAGEN GmBH, Germany) with the RNase-Free DNase Set (QIAGEN). Total RNA was amplified and Cy3-CTP labeled with a Low Input Quick Amp Labeling Kit, One-Color (Agilent). Labeled cRNA was purified with the RNeasy mini kit (QIAGEN). Slides were hybridized with 1.65 µg Cy3-labeled cRNA, processed, and scanned on an Agilent Microarray Scanner (Agilent) with default settings. Data were extracted with Feature Extraction software 10.7 (Agilent). Raw data were normalized using the Quantile algorithm in Gene Spring Software 11.0 (Agilent). The raw and normalized datas were deposited in the NCBI’s Gene Expression Omnibus (GEO) database (Accession NO. GSE58988). Differentially expressed gene identification (FC ≥2, *p*<0.05) and pathway enrichment analysis were performed by the SAS online system of ShanghaiBio Corporation. GO enrichment analysis was performed with the SAS online system and the GOEAST web-based software (http://omicslab.genetics.ac.cn/GOEAST/index.php). The Venn diagram was drawn with R package “VennDiagram”.

### Real-time quantitative PCR

To validate the microarray data, expression of *per1b*, *nr1d1, per2*, *dbp2*, *opn1mw2*, *tmx3,* and *ascl1b* was determined by qPCR analysis. These genes were selected for their microarray-derived expression patterns and based on previous reports on cold stress response. Samples were analyzed in two batches. One batch was the same as that used in the microarray analysis. The second batch included samples from the timed cold treatments (five fish were randomly sampled from each group).

First-strand cDNA was synthesized from 2 µg total RNA using random hexamer primers. PCR primers were designed using Primer Premier 5.0 software (Premier, Canada). qPCR was performed in a Mastercycler ep realplex (Eppendorf). The 20-µL reactions contained 2× SYBR Green Real-Time PCR Master mix (TAKARA), 2 pmol each primer, and 10× diluted cDNA templates. All reactions were performed in triplicate with the following cycling conditions: 40 cycles of 5 s at 95°C, 30 s at 56°C and 20 s at 72°C, followed by melt curve analysis. Reaction specificity was confirmed by the observation of a single melt peak at the expected Tm. The sequences, Tm, and amplification efficiency, accession number, gene name, and amplicon lengths are listed in Table S1.

### Statistical analysis

Swimming status and ATP concentrations were analyzed by one-way ANOVA followed by *t*-test. qPCR results were analyzed by two-way ANOVA followed by Bonferroni post-tests. Significant difference was defined as *P*<0.05. Data represent mean ± S.E.M.

## Results

### A transgenic line expressing M3CK in skeletal muscle

**Figure 2:**
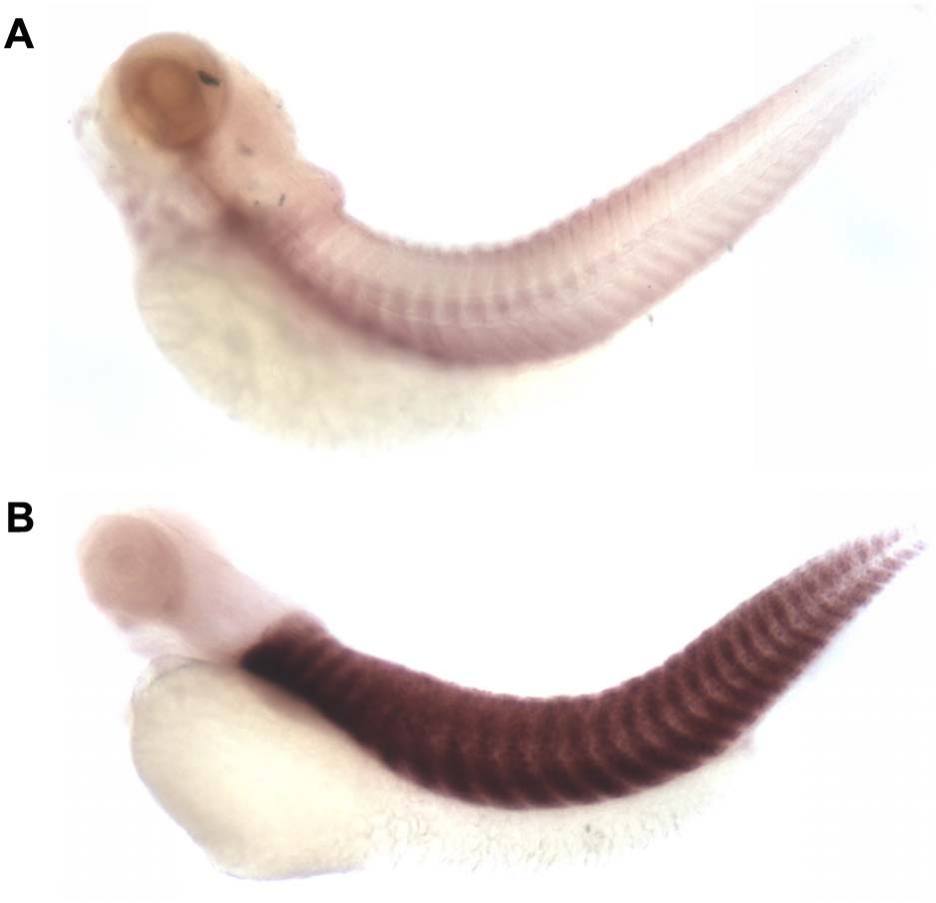
Expression of *m3ck* in transgenic fish by *in situ* hybridization. Whole-mount *in situ* hybridization was used to detect *m3ck* expression in wild-type (A) and *Tg* (*smyd1:m3ck*) fish (B) at 72 hpf. The signal was evaluated by microscopy for color detection of immunoreactive signals. Scale bars = µm.

One out of nine putative founders was found to carry the *smyd1:m3ck* transgene by germ-line transmission and selected to generate the transgenic line (Figure 1B). *In situ* hybridization confirmed that carp *m3ck* was specifically expressed in the skeletal muscle (Figure 2). The carrying rates of the F1 and F2 generation were 16.22% (6/37) and 34.9% (22/63), respectively. The transgenic cassette was proved to be inserted into a non-coding area by inverse PCR.

### Swimming behavior and energy

**Figure 3:**
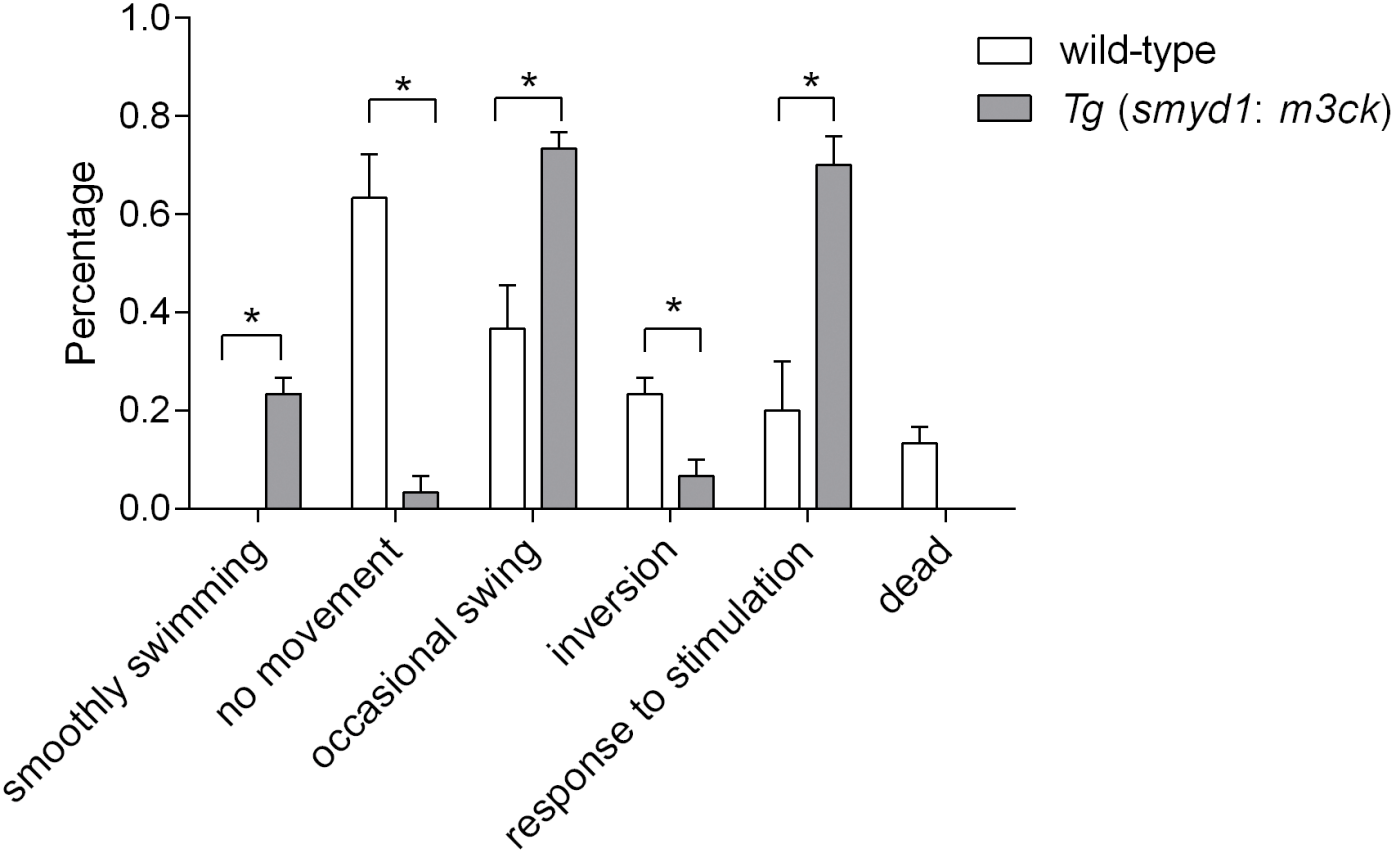
Status of transgenic and non-transgenic fish at 13°C. The percentages smooth swimming, occasional swing, response to external stimulation, no movement, inversion, and dead fish were recorded. Data were analyzed by one-way ANOVA and *t-test* to compare data between fish lines. Data represent mean ± S.E.M. (*n* = 30). “*”indicates *P*<0.05.

During exposure to 13°C water temperature, the wild-type fish instantly lost their swimming ability. They did not move; they were imbalanced, inverted, disoriented, and did not respond to external stimulation such as gentle knocking on the side of the container. In contrast, *Tg* (*smyd1:m3ck*) fish did not move or were inverted for the first 5–10 min, after which normal swimming was restored (Video S1 and Video S2). The percentages of fish exhibiting smooth swimming, occasional swing, and response to external stimulation were 23.3% (7/30), 73.3% (22/30), and 70.0% (21/30), respectively, in *Tg* (*smyd1:m3ck*) fish and 0, 36.7% (11/30), and 20.0% (6/30) in wild-type (*P*<0.05). The percentages of transgenic fish exhibiting no movement [3.3% (1/30)] and inversion [6.7% (2/30)] were lower than in wild-type fish [63.3% (19/30) and 23.3% (7/30); *P*<0.05]. The number of deaths did not significantly differ between groups (Figure 3). Thus, we concluded that our transgenic fish performed better than wild-type under cold stress.

**Figure 4:**
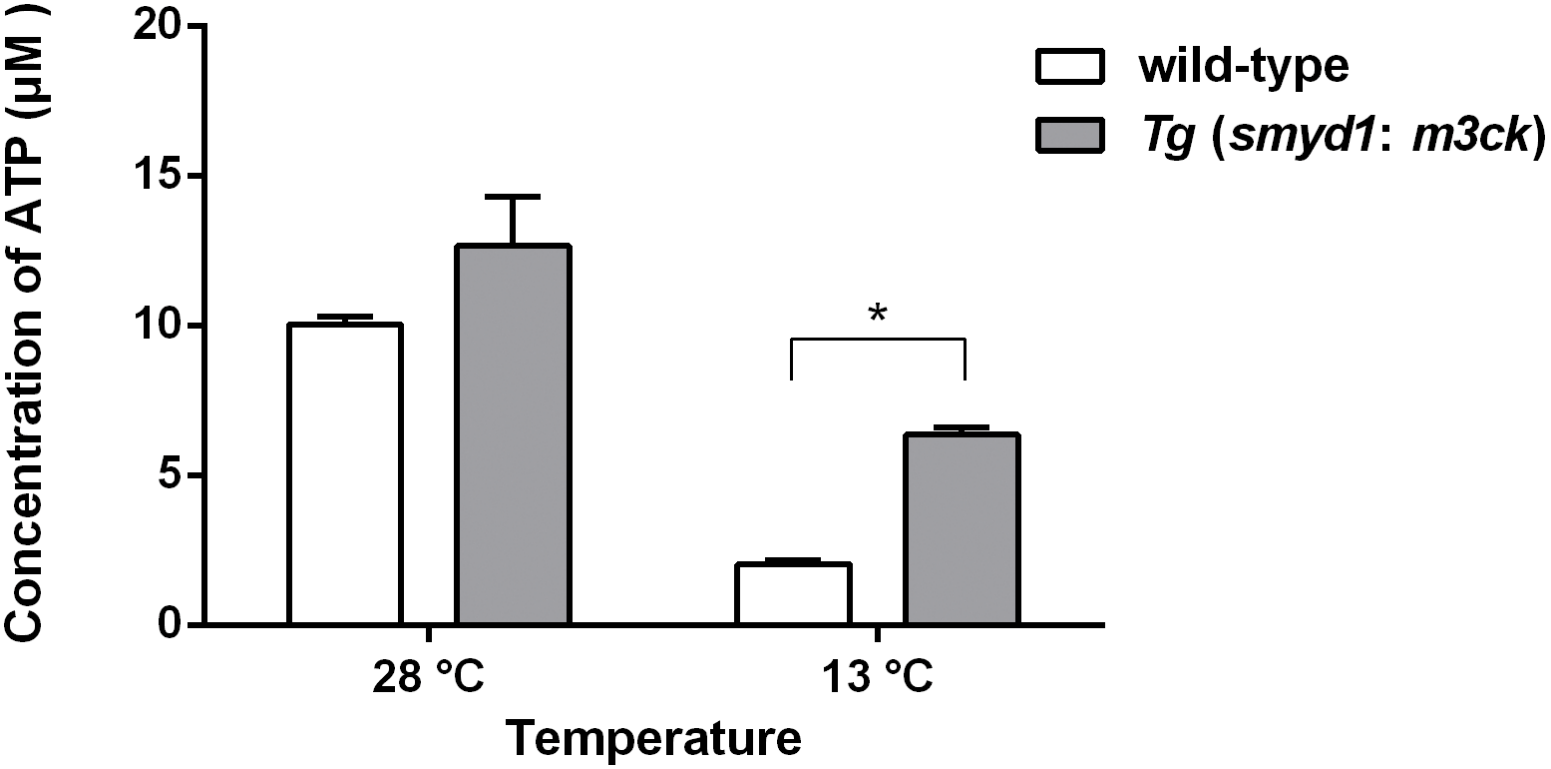
ATP concentration in transgenic and non-transgenic fish at 28°C or 13°C. The concentration of ATP in *Tg* (*smyd1:m3ck*) and wild-type fish was measured at the indicated times. Data were analyzed by one-way ANOVA and *t-test*. Data represent mean ± S.E.M. (*n* = 5). “*”indicates *P*<0.05.

ATP concentrations were 10.04 µM at 28°C and 2.01 µM at 13°C in wild-type fish and 12.66 µM at 28°C and 6.36 µM at 13°C in *Tg* (*smyd1:m3ck*) fish (Figure 4). Thus, the concentration of ATP tended to increase in *Tg* (*smyd1:m3ck*) versus wild-type fish at 28°C. After 2 h at 13°C, the ATP concentration in *Tg* (*smyd1:m3ck*) fish was 2.16-fold greater than in wild-type fish (*P*<0.05). If we define the wild-type fish at 28°C as normal, the ATP concentration at 13°C was 63.3% in *Tg* (*smyd1:m3ck*) fish, but only 20.0% in wild-type fish.

### Microarray analysis

**Figure 5:**
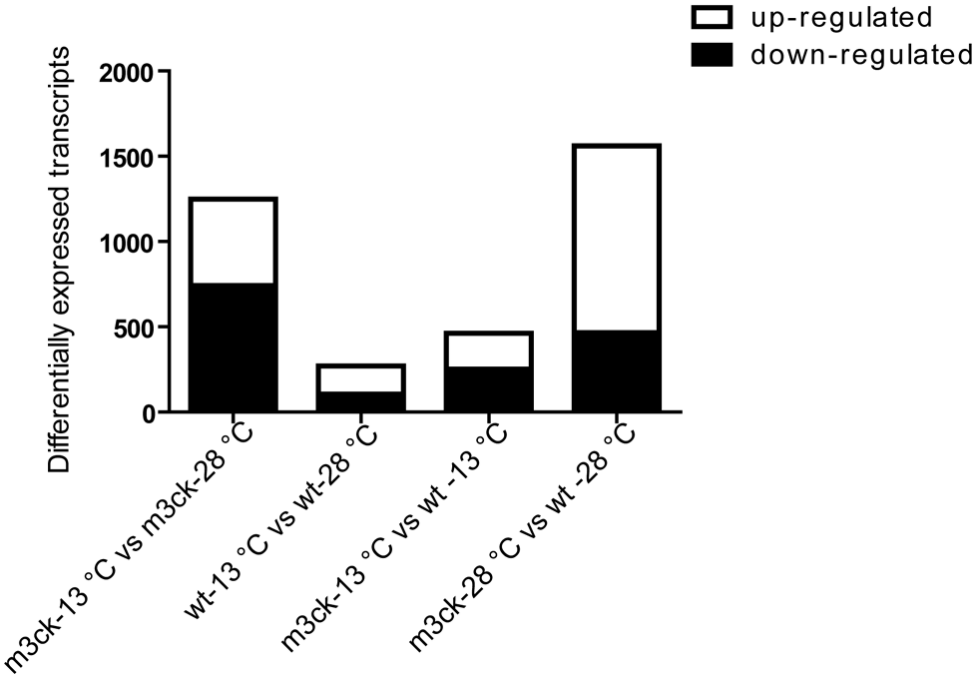
Relative abundance of up- and down-regulated transcripts in the experimental groups. The numbers of differentially expressed genes are shown. Transcripts with fold change ≥2, *P* value <0.05, were considered to be differentially expressed.

Agilent microarray analysis was used to detect whole-body gene expression in transgenic and wild-type fish. Principle Component Analysis (PCA) was performed to verify consistency within groups and differences between groups. There were 507 up-regulated transcripts and 742 down-regulated transcripts in the m3ck-13°C vs. m3ck-28°C comparison. In the wt-13°C vs. wt-28°C comparison, there were 165 up-regulated transcripts and 105 down-regulated transcripts (Figure 5). Under cold stress, there were 210 up-regulated and 252 down-regulated transcripts in *Tg* (*smyd1*:*m3ck*) vs. wild-type fish and at 28°C, there were 1095 up-regulated transcripts and 466 down-regulated transcripts in m3ck-28°C vs. wt-28°C fish.

### Validation by quantitative real-time PCR

To validate the microarray results, relative mRNA levels for seven genes were measured by qPCR (Figure S1). The results showed excellent agreement with the microarray results and confirmed the reliability of the single-channel microarray analysis method.

### Overlapping expressed genes under cold stress

**Figure 6:**
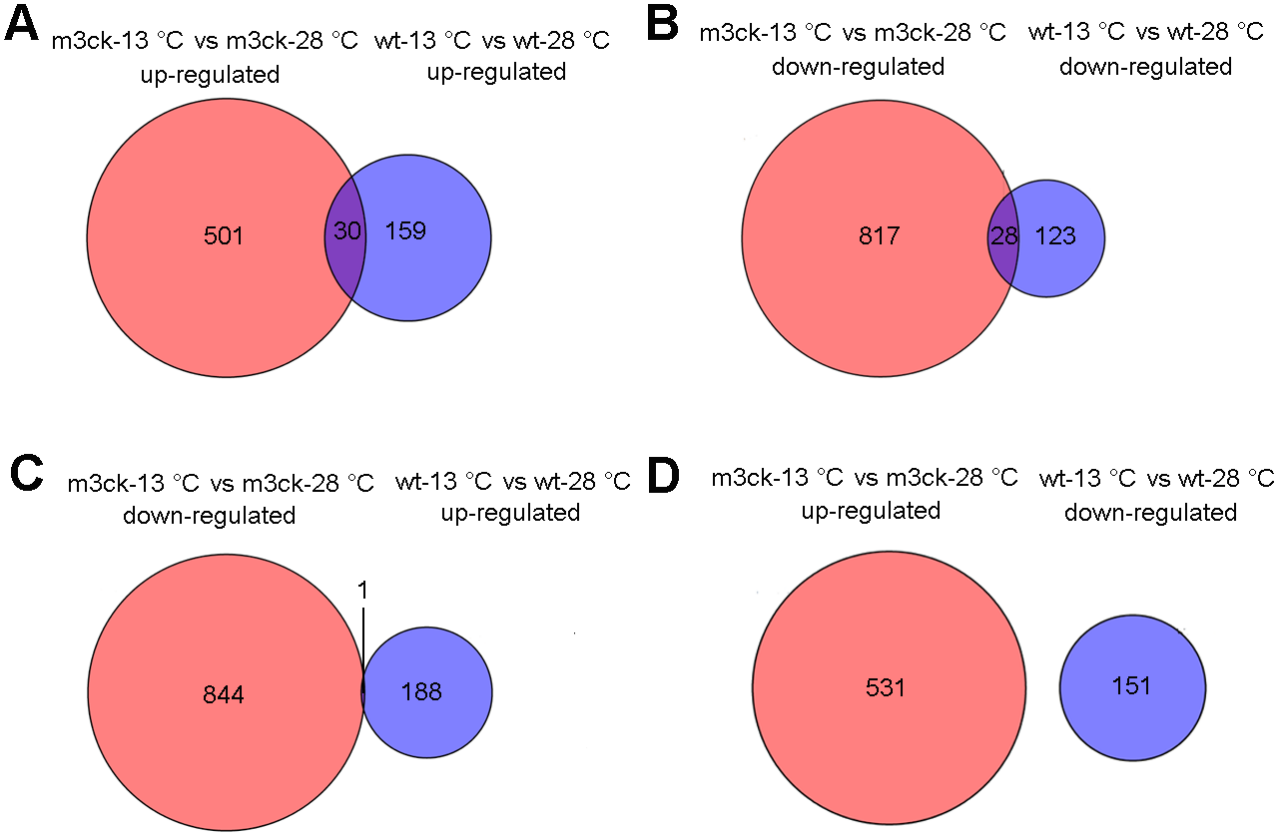
Overlapping expressed transcripts under cold stress. Venn diagrams represent the number of overlapping and differentially expressed genes between A. Up-regulated transcripts under cold stress (2 h) of *Tg* (*smyd1:m3ck*) and wild-type fish. B. Down-regulated transcripts under cold stress of *Tg* (*smyd1:m3ck*) and wild-type fish. C. Down-regulated transcripts under cold stress of *Tg* (*smyd1:m3ck*) and up-regulated transcripts in wild-type fish. D. Up-regulated transcripts under cold stress of *Tg* (*smyd1:m3ck*) and down-regulated transcripts in wild-type fish.

After 2 h under cold stress, more genes were differentially expressed genes in *Tg* (*smyd1:m3ck*) than in wild-type fish (Figure 6). Thirty up-regulated transcripts were shared by m3ck-13°C vs. m3ck-28°C and wt-13°C vs. wt-28°C (Figure 6A), corresponding to *cry1b* (cryptochrome 1b), *cry5* (cryptochrome 5), *cry3* (cryptochrome 3), *nos2a* (nitric oxide synthase 2a, inducible), *illb* (interleukin 1, beta), *per1b* [period homolog 1b (*Drosophila*)], *per2* [period homolog 2 (*Drosophila*)], *egr1* (early growth response 1), *nr1d1* (nuclear receptor subfamily 1, group d, member 1), *dbp2* (D-box binding protein 2), and *slc35f3* (solute carrier family 35, member F3). Twenty-eight genes were down-regulated (Figure 6B), including *opn1mw1* [(opsin 1 (cone pigments), medium-wave-sensitive 1], *opn1lw2* [opsin 1 (cone pigments), long-wave-sensitive 2], *tmx3* (thioredoxin-related transmembrane protein 3), *grk1b* (G protein-coupled receptor kinase 1 b), *nmnat1* (nicotinamide nucleotide adenylyltransferase 1), *trpv6* (transient receptor potential cation channel, subfamily V, member 6), *guca1e* (guanylate cyclase activator 1e), *taar14h* (trace amine associated receptor 14 h), *itpr3* (inositol 1,4,5-triphosphate receptor, type 3), *abcg4b* [ATP-binding cassette, sub-family G (WHITE), member 4b], and *ascl1b* [achaete-scute complex-like 1b (*Drosophila*)]. However, only one transcript was down-regulated in m3ck-13°C vs. m3ck-28°C but up-regulated in wt-13°C vs. wt-28°C: *retinol dehydrogenase 13-like* (Figure 6C). No transcript was found to be up-regulated in m3ck-13°C vs. m3ck-28°C and down-regulated in wt-13°C vs. wt-28°C (Figure 6D).

### Gene Ontology (GO) enrichment analysis

**Figure 7:**
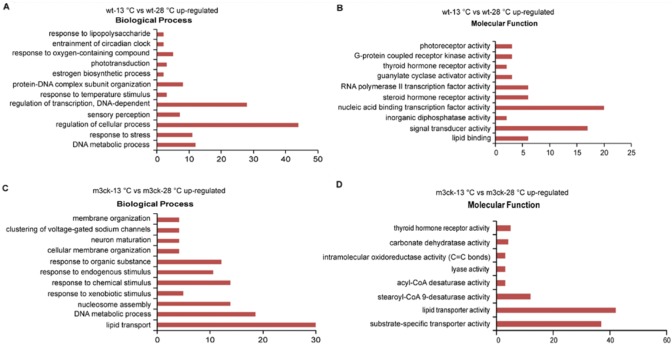
GO enrichment analysis of up-regulated transcripts in transgenic and wild-type fish under cold stress. A. Biological processes of wt-13°C vs. wt-28°C up-regulated genes. B. Molecular functions of wt-13°C vs. wt-28°C up-regulated genes. C. Biological processes of m3ck-13°C vs. m3ck-28°C up-regulated genes. D. Molecular functions of m3ck-13°C vs. m3ck-28°C up-regulated genes. The horizontal axis represents transcript count in each GO term. Up-regulated transcripts were submitted for GO enrichment analysis using the SAS online analysis system.

To identify transcriptional events that occur during cold acclimatization, up- and down-regulated genes in each group were subjected to GO enrichment analysis (Table S2). In wild-type fish, cold stress induced biological processes such as regulation of cellular processes, regulation of DNA-dependent transcription, DNA metabolic process, response to stress, and protein-DNA complex subunit organization (Figure 7A and Figure S2). Up-regulated molecular functions included nucleic acid binding transcription factor activity, signal transducer activity, lipid binding, and thyroid hormone receptor activity (Figure 7B). In transgenic fish, cold-induced biological processes included lipid transport, DNA metabolic process, responses to endogenous stimulus, membrane organization, and clustering of voltage-gated sodium channels (Figure 7C). Among these processes, lipid transport was the most enriched term (Figure S3). Up-regulated molecular functions included lipid transporter activity, substrate-specific transporter activity, and acyl-CoA desaturase activity (Figure 7D).

**Figure 8:**
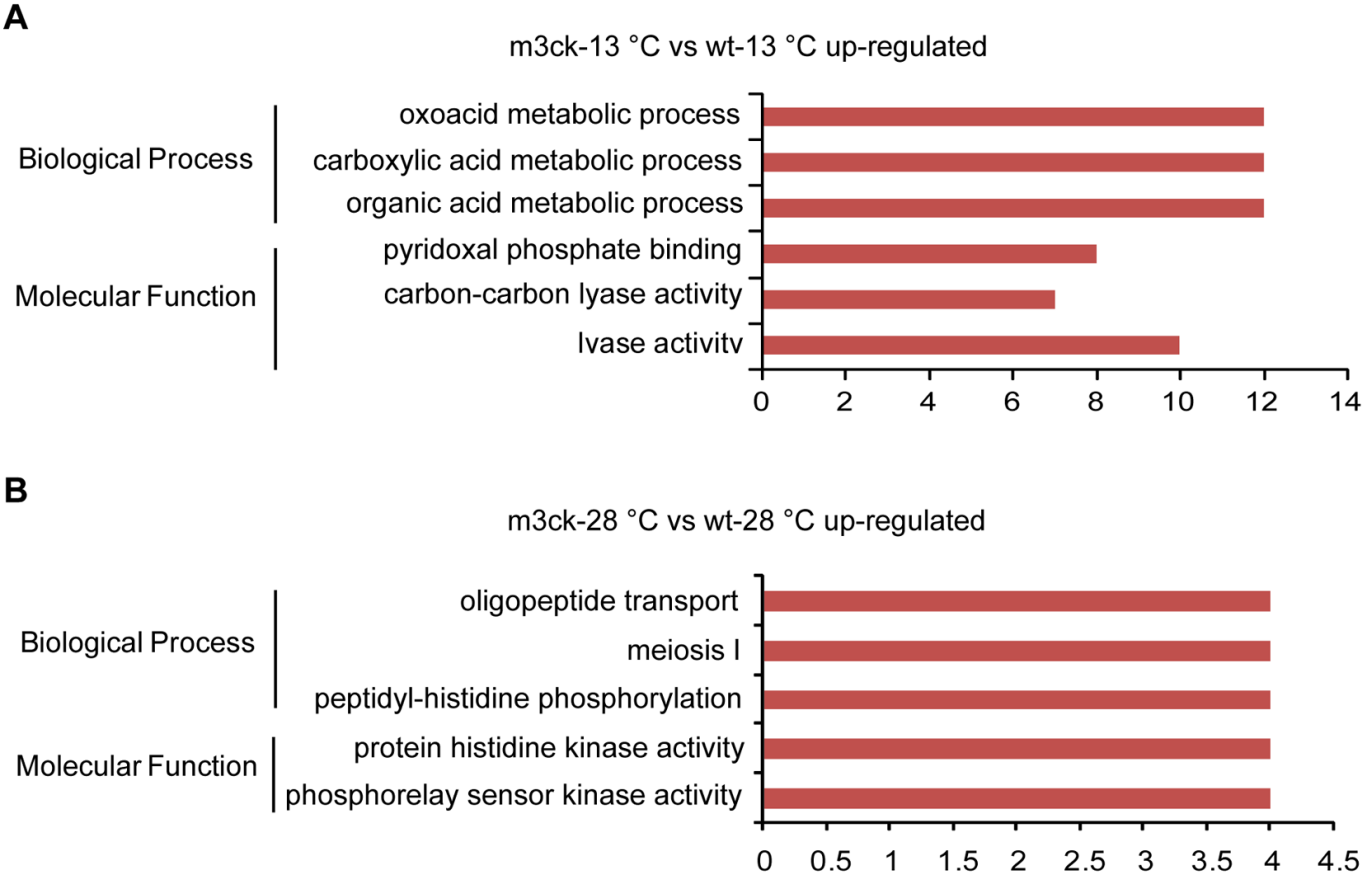
GO enrichment analysis of up-regulated transcripts between transgenic fish and wild-type fish under cold stress or normal temperature. A. m3ck-28°C vs. wt-28°C up-regulated transcripts. B. m3ck-13°C vs. wt-13°C up-regulated transcripts. The horizontal axis represents transcript count in each GO term. Up-regulated transcripts were submitted for GO enrichment analysis using the SAS online analysis system.

Biological processes such as organic acid metabolic process, carboxylic acid metabolic process, and oxoacid metabolic process, were enriched among m3ck-13°C vs. wt-13°C cold stress-induced genes (Figure 8A). At normal temperature, however, overrepresented GO terms among m3ck-28°C vs. wt-28°C up-regulated genes were peptidyl-histidine phosphorylation, oligopeptide transport, and meiosis I (Figure 8B).

**Figure 9:**
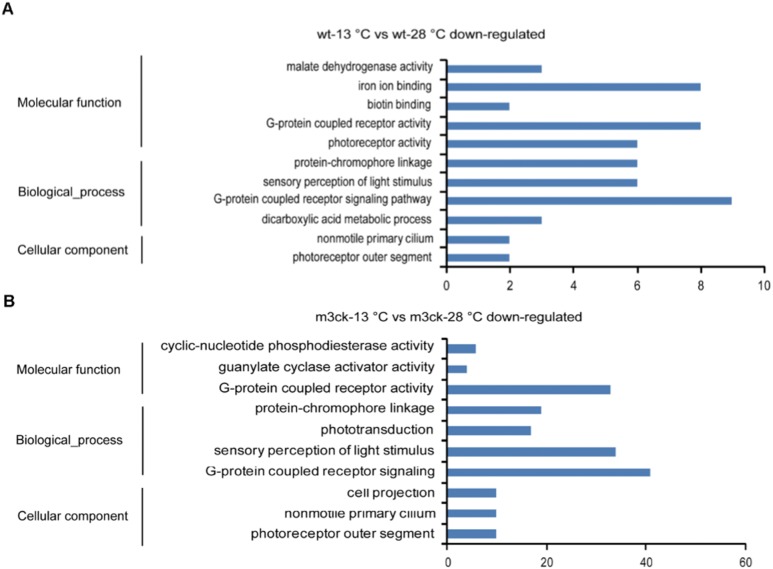
GO enrichment analysis of down-regulated transcripts of transgenic and wild-type fish under cold stress. A. wt-13°C vs. wt-28°C down-regulated transcripts. B. m3ck-13°C vs. m3ck-28°C down-regulated transcripts. The horizontal axis represents transcript count in each GO term. Down-regulated transcripts were submitted for GO enrichment analysis using the SAS online analysis system.

**Figure 10:**
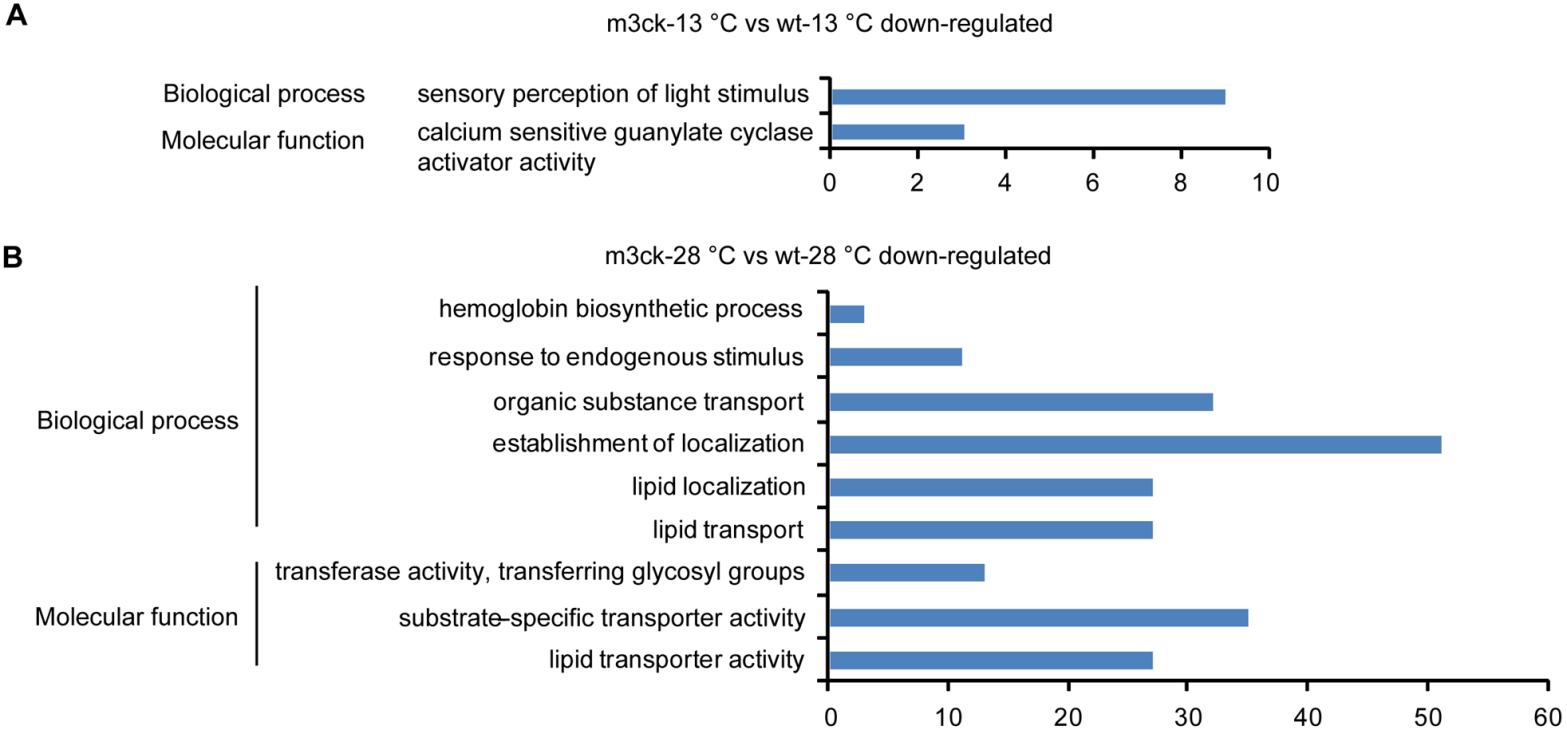
GO enrichment analysis of down-regulated transcripts between transgenic and wild-type fish under cold stress or normal temperature. A. m3ck-28°C vs. wt-28°C down-regulated transcripts. B. m3ck-13°C vs. wt-13°C down-regulated transcripts. The horizontal axis represents transcript count in each GO term. Up- and down-regulated transcripts were submitted for GO enrichment analysis using the SAS online analysis system.

Biological processes inhibited by cold stress in wild-type fish included the G-protein coupled receptor signaling pathway, sensory perception of light stimulus, protein-chromophore linkage, and dicarboxylic acid metabolic process (Figure S4). Molecular functions included G-protein coupled receptor activity, iron ion binding, photoreceptor activity, malate dehydrogenase activity, and biotin binding (Figure 9A). For transgenic fish, cold stress-inhibited biological processes included the G-protein coupled receptor signaling pathway, sensory perception of light stimulus, protein-chromophore linkage, and phototransduction (as in wild-type). Molecular functions included G-protein coupled receptor activity, cyclic-nucleotide phosphodiesterase activity, and guanylate cyclase activator activity (Figure 9B, Figure S5). Biological processes of the sensory perception of light stimulus and molecular function of calcium-sensitive guanylate cyclase activator activity were enriched in m3ck-13°C vs. wt-13°C cold stress-inhibited genes (Figure 10A). At normal temperatures, down-regulated biological processes in m3ck-28°C vs. wt-28°C were the establishment of localization, organic substance transport, and lipid transport. Molecular functions included substrate-specific transporter activity; lipid transporter activity; and transferase activity, transferring glycosyl groups (Figure 10B).

### Pathway enrichment in cold stress-regulated genes

**Figure 11:**
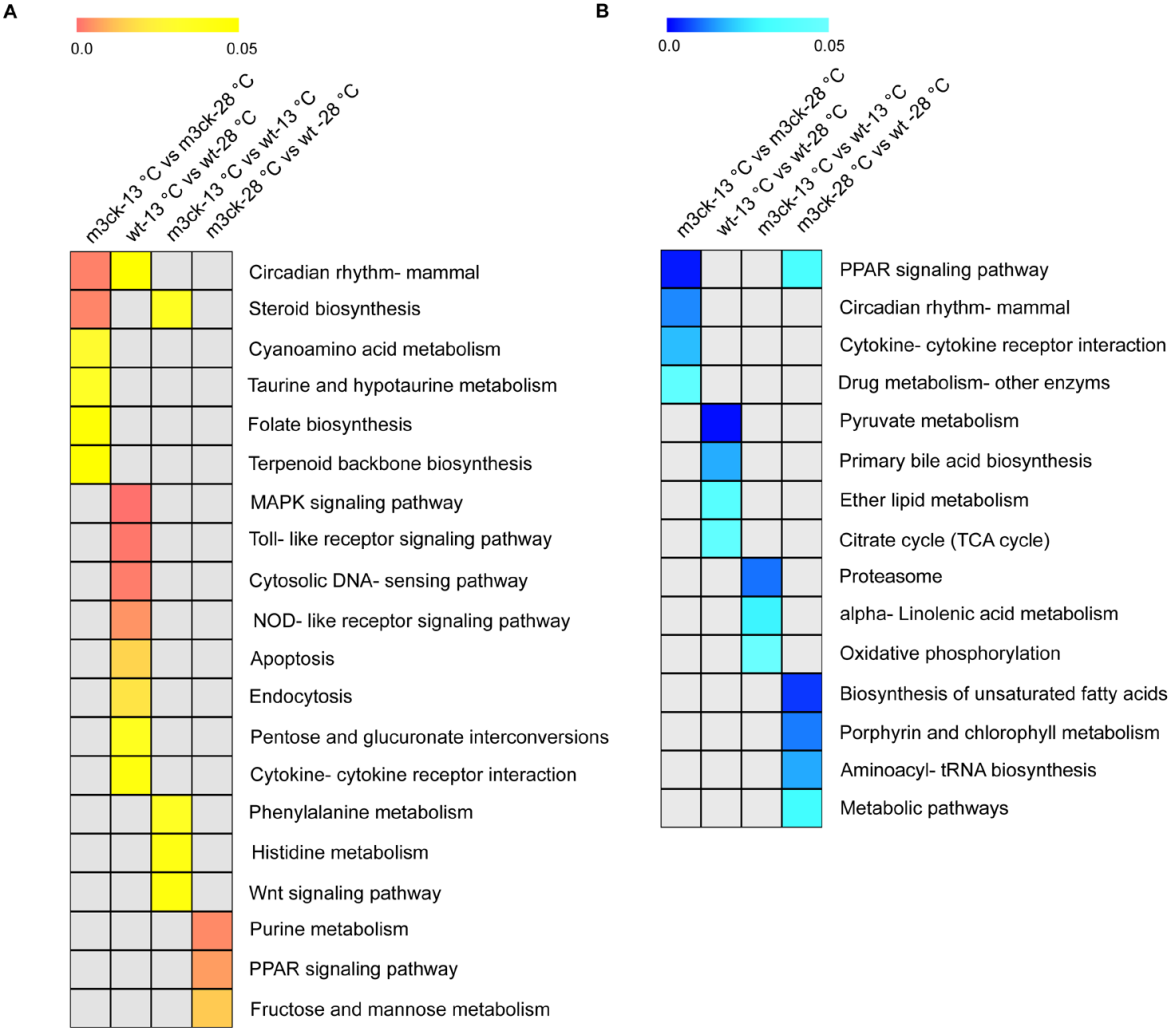
KEGG pathway analysis of cold-regulated genes. A. Up-regulated genes. B. Down-regulated genes. Up- and down-regulated genes were submitted for KEGG enrichment analysis using the SAS online analysis system. Color scales represent *P* values of enrichment tests and gray cells indicate an empty value or *P*>0.05.

In wild-type fish, cold-induced pathways included MAPK signaling, circadian rhythm, cytosolic DNA-sensing, pentose and glucuronate interconversions, and NOD-like receptor signaling, toll-like receptor signaling pathway, and apoptosis. In transgenic fish, cold-induced pathways included the molecular clock, steroid biosynthesis, cyanoamino acid metabolism, taurine and hypotaurine metabolism, folate biosynthesis, and terpenoid backbone biosynthesis. These pathways were mainly involved in the regulation of biological processes such as environmental adaptation, signal transduction, membrane construction, and cell adhesion (Figure 11A, Table S3). In addition, cold stress inhibited primary bile acid biosynthesis, pyruvate metabolism, ether lipid metabolism, and citrate cycle (TCA cycle) in wild-type fish. Some cold-inhibited pathways such as cytokine-cytokine receptor interaction, the PPAR signaling pathway, circadian rhythm, and drug metabolism were overrepresented in transgenic fish (Figure 11B, Table S3). It is important to note that some enriched pathways or GO terms may not reflect actual situations in some tissues or cells due to our use of whole organisms for this study.

## Discussion

In this study, we established a transgenic zebrafish line *Tg* (*smyd1:m3ck*) with energy-producing related genes to investigate the mechanism of cold tolerance. Microarray analysis revealed numerous genes, key biological processes, and intracellular pathways associated with cold acclimation. These results might offer insights into the molecular mechanisms underlying gene expression during cold stress in fish.

To survive in the wild, zebrafish must adapt to changes in water temperature. Daily variations of water temperature in the Ganges basin, a habitat of wild zebrafish, were recorded as ranging from a low of ±0.1°C to a high of ±5.6°C, with seasonal variations of 15.0±0.4°C. The lowest recorded water temperature in a zebrafish habitat was 16.5°C, the highest was 38.6°C [19]. Several factors influence thermal tolerance in fishes; however, the initial temperature extremity may influence tolerance of temperature shifts regardless of sex [28], [29]. Zebrafish maintained under variable temperature conditions exhibit greater tolerance than fish maintained at constant temperature [30]. These observations suggest pure zebrafish lines in laboratory culture under constant temperature conditions (28°C) may exhibit different tolerances than wild zebrafish. Therefore, we used wild-type strains of zebrafish to study cold temperature tolerance.

We analyzed gene expression profiles in transgenic and wild-type fish at 28°C to detect the impact of the transgene insertion on general metabolism and physiology. Pathways such as purine metabolism, PPAR signaling, and fructose and mannose metabolism were up-regulated in transgenic versus wild-type fish. The up-regulation of purine metabolism may be due to physiological adjustments to remove excess ATP generated by the creatine kinase transgene. PPARs are the major regulators of lipid and fatty acid metabolism and regulate transport, oxidation, storage, and synthesis of fatty acids [31]. Moreover, PPAR activation can enhance sugar metabolism, which may contribute to the up-regulation of fructose and mannose metabolism. Pathways such as biosynthesis of unsaturated fatty acids, aminoacyl-tRNA, and other metabolic pathways were down-regulated in transgenic versus wild-type fish. These genes included *acox3* (acyl-coA oxidase 3), *scd* (stearoyl-CoA desaturase), and *mars* (methionyl-tRNA synthetase). *Acox3* and *scd* mediate fatty acid oxidation [32], [33] and down-regulation might indicate that the overall energy supply in the transgenic fish was sufficient to alleviate the need for fatty acid metabolism. This assumption could be linked to up-regulation of the PPAR signaling pathway, which mediates fatty acid storage. Mars plays a critical role in protein biosynthesis by charging tRNAs with their cognate amino acids [34]. Down-regulation of this process in transgenic fish might also due to the sufficient energy supply and the reduced need for translation of many metabolic enzymes. In comparison to wild-type fish, the transgenic fish used different metabolic strategies to maintain energy homeostasis. In addition, there was no difference observed in transgenic fish versus wild-type fish with and without cold stress (Figure 11). This would suggest that the differential pathways detected were not affected by the transgene manipulate.

A common response to stimuli and the initiation of defensive compensatory mechanisms has been detected in transgenic and non-transgenic fish under cold stress. Under cold-stress, transgenic and non-transgenic fish down-regulate *opsin 1* (cone pigments), *opn1mw1*, *opn1mw4*, *opn1mw2*, and *opn1lw2*. These are rod cell photoreceptors belonging to the G-protein coupled transmembrane receptor family that mediates transmission of optical signals to the brain [35] and thus suggest the importance of the neuronal signal transduction and central nervous system (CNS). It is well known that the CNS of both mammals and fish play important roles in thermoregulation. The hypothalamus is the most notable central thermoregulatory site to trigger the primary response to cold and helps to increase sympathetic output along with the spinal cord to peripheral tissues such as muscle and brown fat [36]–[38].

Up-regulated genes in transgenic and non-transgenic fish under cold stress included GO terms for DNA-dependent negative regulation of transcription and response to light stimulus. These transcripts included the cryptochrome family (*cry-dash*, *Cry 3* and *Cry 5*) and *period homolog* gene family (*per1b* and *per2*). Up-regulation of these genes leads to inhibition of the *clock* gene, the core of the circadian rhythm pathway, and thus down-regulation of the remainder of the pathway. A correlation between temperature and circadian rhythm has been reported in zebrafish [27] and mice [39]; this was considered a “temperature compensation” mechanism to correct the natural tendency of the rate of biochemical reactions to change with temperature [40].

Lipid transport and metabolism-related genes such as *scd* (stearoyl-CoA desaturase, delta-9-desaturase), *sgms2* (sphingomyelin synthase 2), *fdft1* (squalene synthetase), and *ebp* (sterol isomerase) were only up-regulated in transgenic fish. The pathways involving these genes included the biosynthesis of unsaturated fatty acids, sphingolipid metabolism, and steroid biosynthesis. Among these, *scd* mediates biosynthesis of unsaturated fatty acids. Delta-9 catalyzes the synthesis of monounsaturated fatty acids (18∶1 n-9 and 18∶2 n-6), which are required for triglyceride energy storage and contributes to the maintenance of cell membrane fluidity [41]–[43]. Genes such as *fdft1* and *ebp* participate in steroid biosynthesis and bile acid biosynthesis. The activity of *fdft1* could provide precursors to cholesterol synthesis. Cholesterol is an essential structural component of cell membranes and serves as a precursor for the synthesis of steroid hormones such as bile salts, which are important in fat absorption [44]. *Sgms2* is associated with sphingolipid metabolism. Sphingolipids are enriched in the plasma membrane and are important structural lipids. They participate in the formation of membrane domains with cholesterol and affect the distribution and function of membrane proteins. Some less abundant sphingolipids are important modulators of cell signaling [45]–[47]. Thus, our results suggest transgenic fish could maintain their cell membrane construction and fluidity under lower temperatures, unlike the wild-type fish. Cell membrane integrity and fluidity are essential for material transport and cell communication. Cell membrane construction-related pathways such as lipid metabolism and primary bile acid biosynthesis were also down-regulated in wild-type fish under cold stress. Therefore, cell membrane fluidity may be important for low-temperature tolerance in fish.

Our results demonstrated the importance of energy for fish during cold stress. ATP is produced by photophosphorylation, cellular respiration, and fermentation and is involved in many cellular processes, including biosynthetic reactions, motility, and cell division [48]. The concentration of ATP in the transgenic fish at low temperatures was 60% (6.36/10.04) of that of wild-type fish at normal temperatures, which was three times (6.36/2.01) that of wild-type fish at low temperatures. This confirmed that M3CK maintains its activity and catalyzes the formation of ATP at low temperatures. Some of this energy may be used for cellular processes such as cell membrane construction in response to low temperature, as indicated by the up-regulation of genes participating in steroid biosynthesis, folate biosynthesis, and terpenoid backbone biosynthesis only in the transgenic fish (Figure 10). It should be noted that the transcripts of genes involved in oxidative phosphorylation, the main source of ATP, were significantly down-regulated in the transgenic fish. However, pathways that lie upstream of oxidative phosphorylation, such as pyruvate metabolism and the TCA cycle, which produce substrates for oxidative phosphorylation, were down-regulated only in wild-type fish at low temperature. Therefore, oxidative phosphorylation could not supply enough energy in response to cold stress. It was interesting to note that the transcripts of genes involved in pentose and glucuronate inter-conversions were up-regulated in wild-type fish under cold stress. This is another metabolic pathway to producing glucose and ribose-5-phosphate (R5P), which could be used in the synthesis of nucleotides and nucleic acids. Therefore, glucose metabolism cannot be the main supplier of energy, and other energy-producing pathways are more important during cold stress. Further studies on the supply of energy should be performed.

Transcripts of the *jun* and *fos* genes were found only in cold treated wild-type fish. They were also up-regulated in previous studies in zebrafish [49] and rats [50], [51] under cold stress. Fos can form heterodimers with Jun, forming a transcription factor complex that often induces apoptosis [52], [53] under stress, and is important for maintaining cell homeostasis. During cold stress, cell damage may trigger the immune response, and many genes involved in immune response pathways were significantly up-regulated in cold-treated wild-type fish, such as Toll-like receptor signaling [54], cytosolic DNA-sensing [55], NOD-like receptor signaling [56], and cytokine-cytokine receptor interactions [57] (Figure 10). Genes involved in the synthesis of nucleotides and nucleic acid-pentose and glucuronate inter-conversions were up-regulated in wild-type fish under cold stress (Figure 10). During the immune response, apoptosis functions as a defense reaction to remove damaged cells; however, the genes involved in cytokine-cytokine receptor interactions were down-regulated in transgenic zebrafish under cold stress. Thus, injury may cause cell death at low temperatures and injury might be another important concern for fish under cold stress.

In conclusion, we identified multiple biological processes, intracellular signaling pathways, and key genes associated with cold tolerance by using muscle-specific transgenic zebrafish. The regulation of the energy metabolic process, cell membrane construction and fluidity, and cell death were important for acclimation to low temperatures. Our results also suggest energy is a critical factor in low-temperature adjustment. For further validation of our hypothesis, protein changes under cold stress will be a very interesting experiment that we plan to pursue in our future work, in addition to a study of metabolite profiles.

## Supporting Information

Figure S1 **Validation of microarray data by real-time quantitative PCR.** qRT-1 represented qPCR with the same samples used in the microarray. qRT-2 represented qPCR with the samples cold treated at different times (n = 5). Data of qPCR referred to the left axis of ordinate and data of microarray analysis referred to the right axis of ordinate.
(TIF)

Figure S2 **Biological processes of m3ck-13°C vs. m3ck-28°C up-regulated transcripts.**
(PDF)

Figure S3 **Biological Processes of wt-13°C vs. wt-28°C up-regulated transcripts.**
(PDF)

Figure S4 **Biological processes of m3ck-13°C vs. m3ck-28°C down-regulated transcripts.**
(PDF)

Figure S5 **Biological Processes of wt-13°C vs. wt-28°C down-regulated transcripts.**
(PDF)

Table S1 **qRT-PCR primers used for validating microarray data.**
(XLS)

Table S2 **GO enrichment analysis of cold-regulated genes.**
(XLS)

Table S3 **Pathways enrichment analysis of cold-regulated genes.**
(XLS)

Video S1 **Swimming behavior of wild-type zebrafish at 13°C.** The timeline represents: 00∶00–00∶27: After the fish moved into cold water bath and the beginning of cold treatment; 00∶28–01∶02: after cold treated for 1 h; 01∶03–01∶36: after cold treated for 2 h.
(MPG)

Video S2 **Swimming behavior of** ***Tg*** **(** ***smyd1:m3ck*** **) zebrafish at 13**°C**.** The timeline represents: 00∶00–00∶26: After the fish moved into cold water bath and the beginning of cold treatment; 00∶27–01∶00: after cold treated for 1 h; 01∶01–01∶31: after cold treated for 2 h.
(MPG)
