# Supplementary figures and images for: Analyzing Cold Tolerance Mechanism in Transgenic Zebrafish (Danio rerio)
Source: PLoS One. 2014 Jul 24;9(7):e102492. doi: 10.1371/journal.pone.0102492 (PMC4109919; doi:10.1371/journal.pone.0102492)

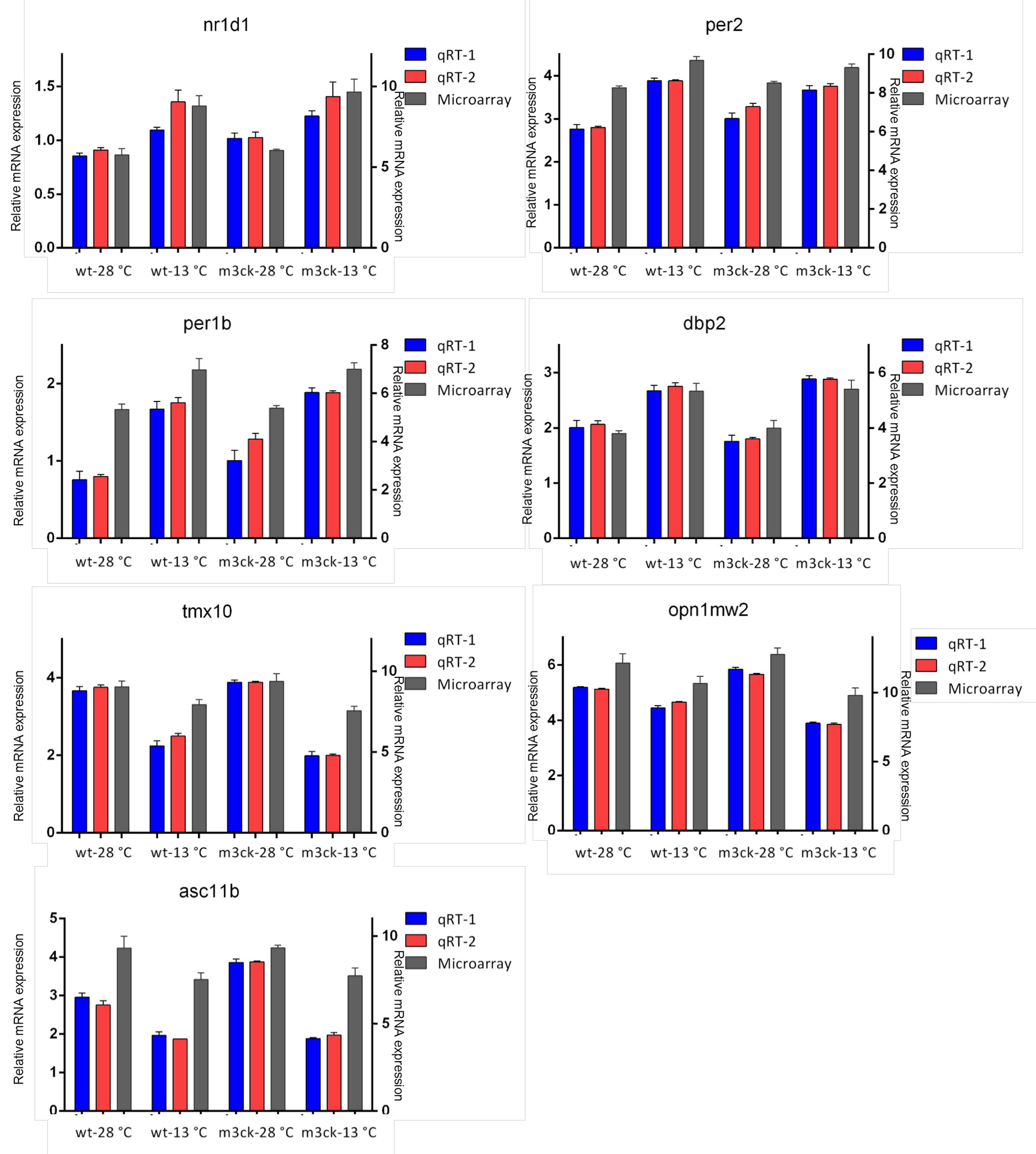

Supplement: Figure S1 — Validation of microarray data by real-time quantitative PCR. qRT-1 represented qPCR with the same samples used in the microarray. qRT-2 represented qPCR with the samples cold treated at different times (n = 5). Data of qPCR referred to the left axis of ordinate and data of microarray analysis referred to the right axis of ordinate. (TIF) [file pone.0102492.s001.tif]

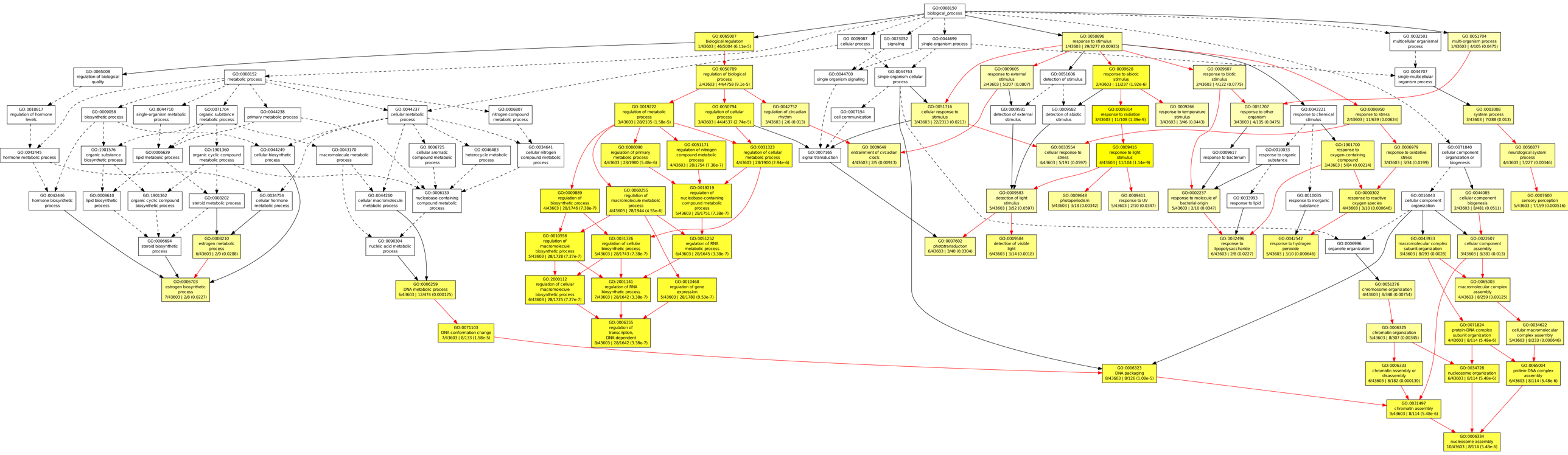

Supplement: Figure S2 — Biological processes of m3ck-13°C vs. m3ck-28°C up-regulated transcripts. (PDF) [file pone.0102492.s002.pdf]

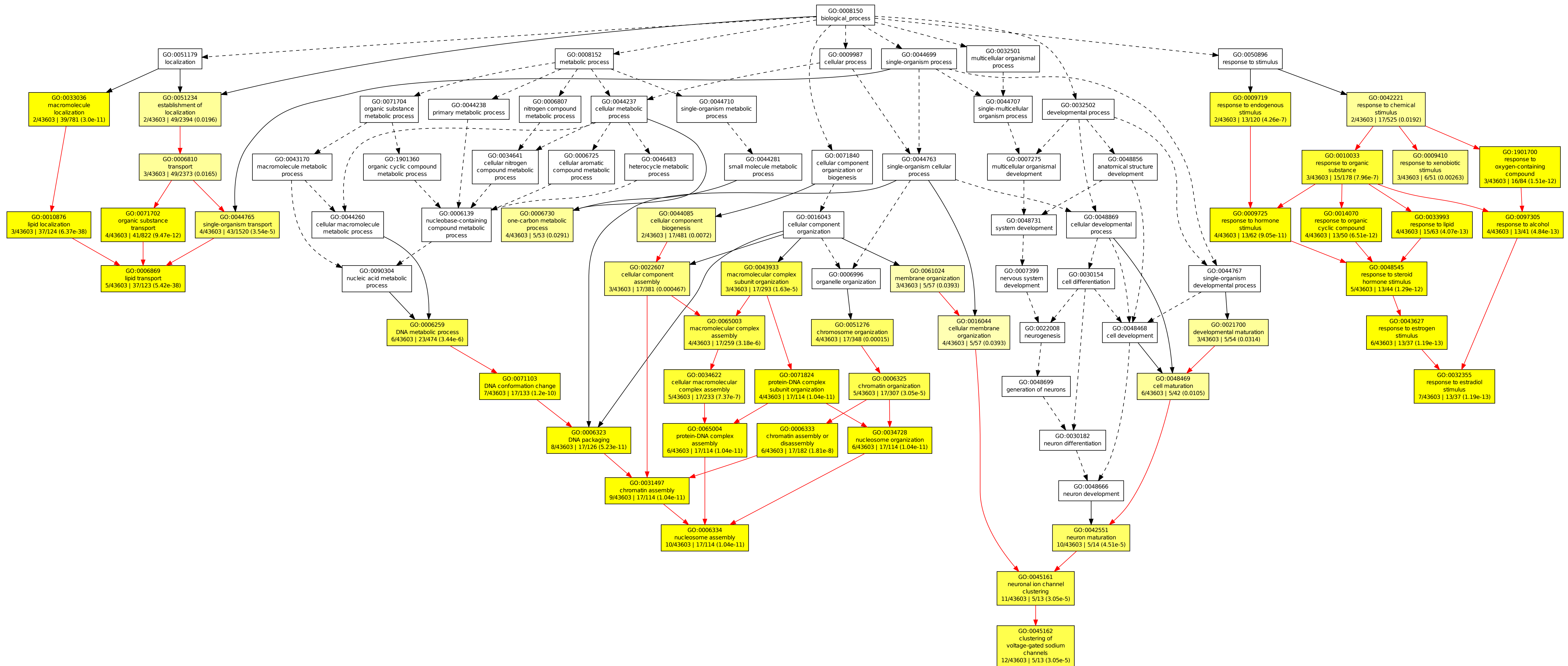

Supplement: Figure S3 — Biological Processes of wt-13°C vs. wt-28°C up-regulated transcripts. (PDF) [file pone.0102492.s003.pdf]

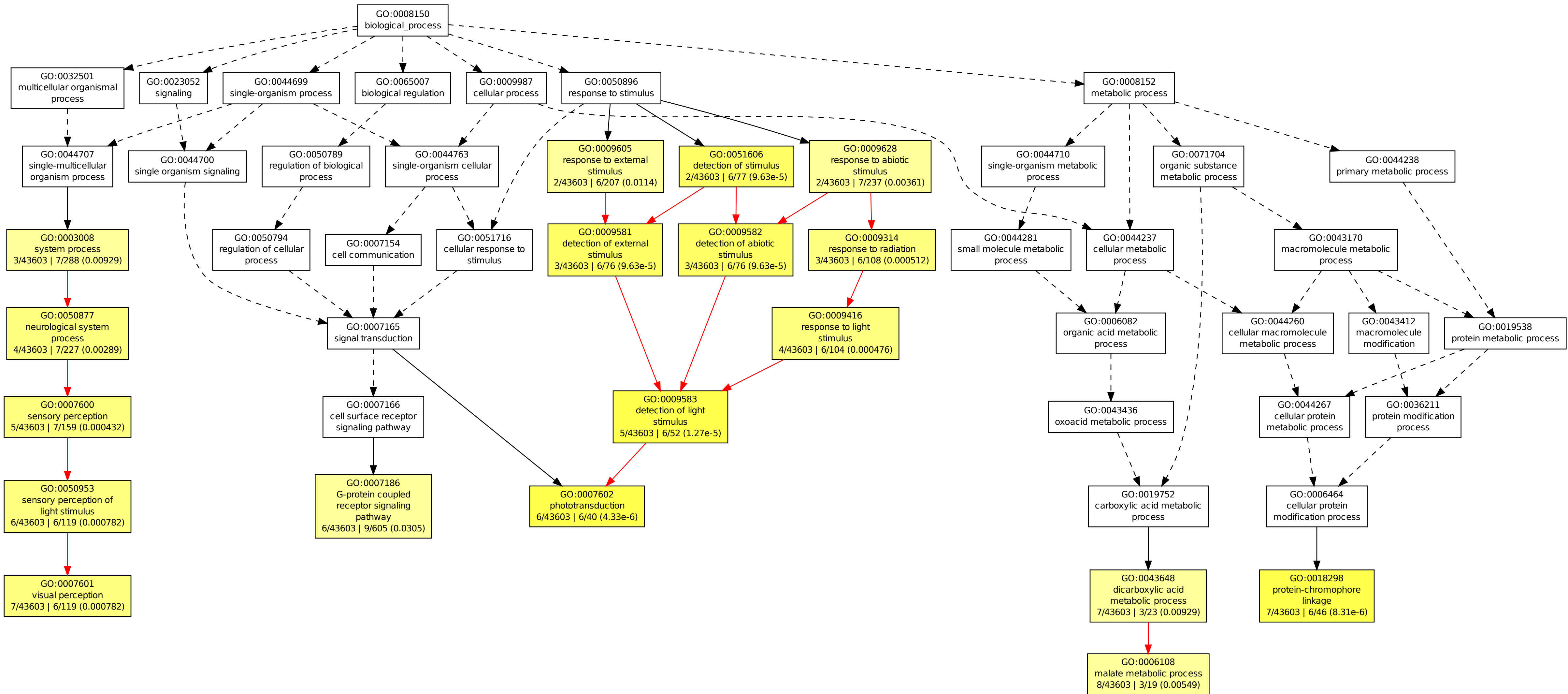

Supplement: Figure S4 — Biological processes of m3ck-13°C vs. m3ck-28°C down-regulated transcripts. (PDF) [file pone.0102492.s004.pdf]

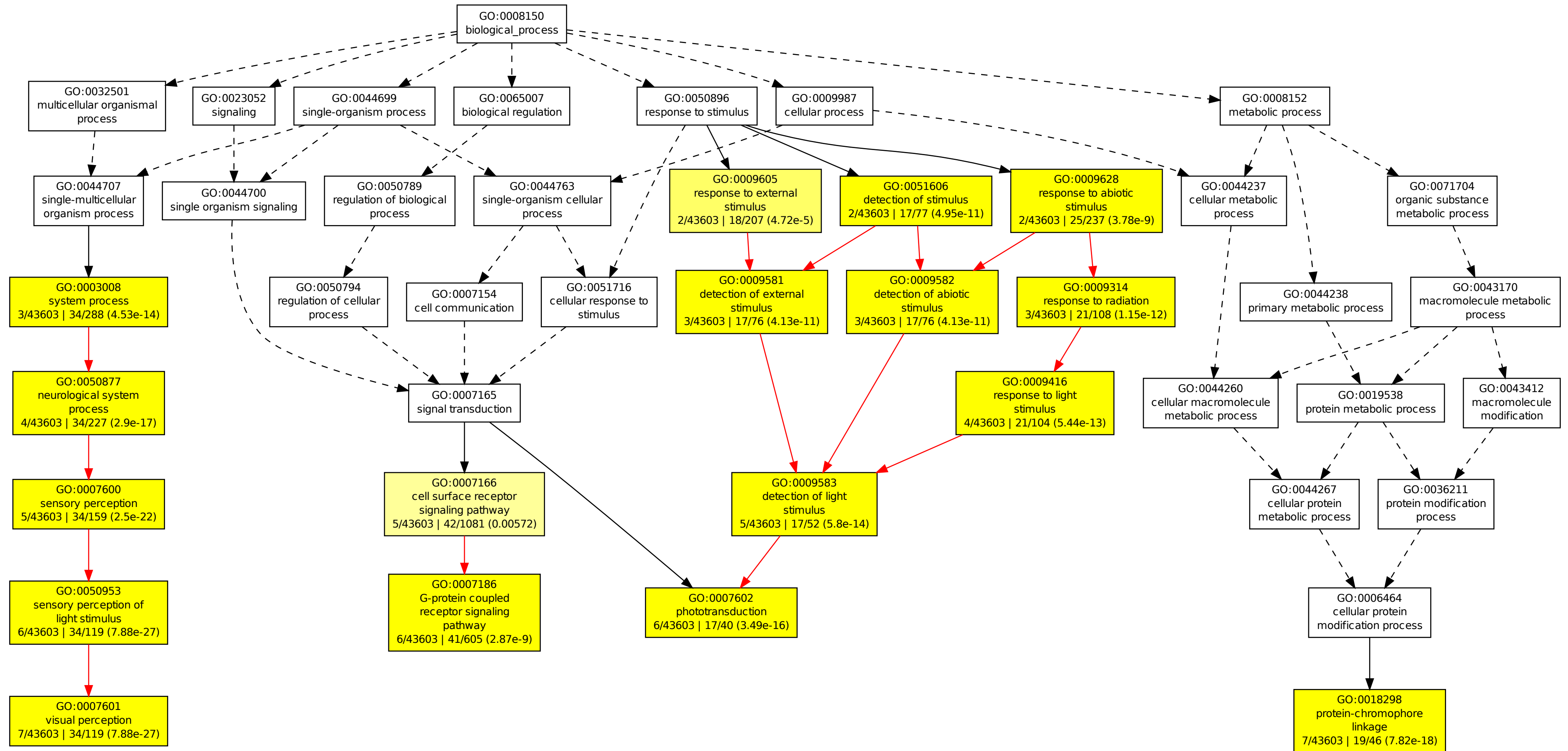

Supplement: Figure S5 — Biological Processes of wt-13°C vs. wt-28°C down-regulated transcripts. (PDF) [file pone.0102492.s005.pdf]
